# Oral health-related quality of life among 11–12 year old indigenous children in Malaysia

**DOI:** 10.1186/s12903-019-0833-2

**Published:** 2019-07-15

**Authors:** Eizatul Aishah Berhan Nordin, Lily Azura Shoaib, Zamros Yuzadi Mohd Yusof, Nor Malina Manan, Siti Adibah Othman

**Affiliations:** 1 Department of Paediatric Dentistry and Orthodontics, Faculty of Dentistry, University of Malaya, 50603 Kuala Lumpur, Malaysia; 2 Department of Community Oral Health & Clinical Prevention, Faculty of Dentistry, University of Malaya, 50603 Kuala Lumpur, Malaysia

**Keywords:** Orang Asli, Child-OIDP, Oral health, Quality of life, Malaysia

## Abstract

**Background:**

Poor oral health among Malaysian indigenous *Orang Asli* (OA) children may impact on their daily performances.

**Aim:**

To assess the oral health status, related behaviours, and oral health-related quality of life (OHRQoL) among OA children in Cameron Highlands (CH), Malaysia, and to identify the predictor(s) for poor OHRQoL.

**Design:**

This was a cross-sectional study involving 249, 11–12 year old OA children from 4 OA primary schools in CH. The children completed a self-administered questionnaire comprising information on socio-demographics, oral health-related behaviours, and the Malay Child Oral Impacts on Daily Performances (Malay Child-OIDP) index followed by an oral examination. Data were entered into the SPSS version 23.0 software. Non-parametric tests and multiple logistic regression were used for data analysis.

**Results:**

The response rate was 91.2% (*n* = 227/249). The prevalence of caries was 61.6% (mean DMFT = 1.36, mean dft = 1.01) and for gingivitis was 96.0%. Despite the majority reported brushing their teeth ≥ 2x/day (83.7%) with fluoride toothpaste (80.2%), more than two-thirds chewed betel nut ≥ 1/day (67.4%). Majority of the children (97.8%) had a dental check-up once a year. Nearly three-fifths (58.6%) reported experiencing oral impacts on their daily performances in the past 3 months (mean score = 5.45, SD = 8.5). Most of the impacts were of “very little” to “moderate” levels of impact intensity with 90.2% had up to 4 daily performances affected. Most of the impacts were on eating (35.2%), cleaning teeth (22.0%) and relaxing activities (15.9%). Caries in primary teeth is associated with oral impacts among the OA children.

**Conclusions:**

The 11–12 year old OA children in Cameron Highland had high prevalence of caries and gingivitis with the majority chewed betel nut regularly. Caries in primary teeth is associated with poor OHRQoL. Future programmes should target younger age group children to promote positive oral hygiene practices, reduce caries, and improve quality of life.

## Background

*Orang Asli* (OA) refers to the minority indigenous people in Peninsular Malaysia who represent a mere 0.6% of the total population. They tend to reside in remote communities and avoid contacts with outsiders [1]. With regards to oral health, the indigenous people tend to have poor access to curative, preventive and promotive oral health services compared with the general population. They also tend to have high prevalence of dental caries, periodontal disease and tooth loss resulting in significant oral health problems [2].

Poor oral health can profoundly impact on the quality of life. Children who suffer from dental pain, dental abscess, gum disease and damaged teeth may become distress. Subsequently, this may lead to negative impacts on their social, functional and psychological well-being such as difficulties in eating, playing, socialising, and sleeping as well as facing a higher risk of hospitalisation with high treatment costs [3]. School performance would also be affected [4].

Since most oral diseases and their consequences may interfere with daily life performances, it is important to consider the disruptions of physical, psychological and social functioning due to poor oral health when assessing oral health need [5]. Traditionally, clinical parameters have been used to estimate oral health need. Although these measures are important, they mainly diagnose the clinical aspects of disease without consideration of its impact on the quality of life. In order to overcome this problem, the socio-dental indicators or oral health-related quality of life (OHRQoL) instruments have been developed to assess the impact of oral disease on the quality of life [6–8]. These instruments are often used alongside clinical indices for a comprehensive oral health needs assessment.

Many socio-dental measures have been developed to assess OHRQoL in various age groups. The Child Oral Impacts on Daily Performances (Child-OIDP) index is one of the most commonly used instruments to assess OHRQoL in children [9]. It was developed in 2004 and has been shown to be valid and reliable to assess OHRQoL among 12-year-old Thai children [9]. Since then, it has been translated into different languages to measure OHRQoL in schoolchildren in different countries [10–14].

Published studies on OA children’s oral health in Malaysia are still very limited. A study in 1987 found that periodontal disease was present in 25.1% (15.7% had Community Periodontal Index (CPI) Score 1, 9.4% had CPI Score 2) of an OA population sample aged 6–15 years [15]. Another study conducted in the state of Selangor in 1990 showed that caries prevalence in the primary and permanent teeth of 6–15 year old OA children was 87.3 and 53.5%, respectively [16]. Despite of high oral disease burden, no study has been conducted to assess the impact of oral health on OA children’s quality of life. Such information is essential as it would complement the clinical data and provide a comprehensive record on the oral health need assessment of OA children. It may also help to inform decision-making and influence policy-makers to deliver regular oral health-related programmes and services to OA communities. Our study was the first study conducted to assess the impact of oral health on the OHRQoL of OA children in Malaysia. The objectives of this study were to assess the oral health status, oral health behaviours, and OHRQoL among 11–12 year old OA children in Cameron Highlands (CH), Malaysia, and to identify factors associated with their OHRQoL.

## Methods

A cross-sectional study targeting 11–12 year old OA primary schoolchildren in the Pahang state, Peninsular Malaysia was conducted in November 2014. The state was chosen because it accommodates the highest number of OA population in Peninsular Malaysia. CH district in the Pahang state was chosen as the study location because it accommodates 4 primary schools with 100% OA children enrolment. The schools provided boarding facilities for OA children during weekdays.

Sample size calculation was based on oral impact prevalence of 66.7% among 11–12 year old non-OA children in Selangor state, Malaysia assessed using the Malay Child-OIDP index [17], 80% power, and 5% Type 1 error to give a sample size of 342 children. The inclusion criteria were 11–12 year old OA children attending OA schools who could read and write in the Malay language, and whose parents were of OA descendants. Uncooperative children and those absent on data collection day were excluded. Based on the enrolment, the total number of 11–12 year old OA children in the four schools was 249. Therefore, it was decided that all the 249 OA schoolchildren were included in the study.

The questionnaire used in this study comprised three sections; children’s socio demographic information, children’s oral health-related behaviours, and the Malay Child-OIDP index. The oral health-related behaviours section comprised items on toothbrushing frequency, toothpaste use, cigarette smoking, betel nut chewing, and mouth rinsing after meal. Each item was scored on an 8-point rating scale, i.e. ‘more than 3 times a day’, ‘2-3 times a day’, ‘once a day’, ‘2-3 times a week’, ‘once a week’, ‘once a month’, ‘once in 2-12 months’, and ‘never’.

The Child-OIDP index was used in this study as it was relatively short, self-administered, easy to use, and relevant to 11–12 year old children. Its theoretical framework was derived from modifications of the WHO’s International Classification of Impairment, Disabilities and Handicaps [18]. Therefore, it enables measurement of OHRQoL dimensions including oral health impairments, functional limitations and disability [19], which are applicable to assess oral impacts in children. The Malay Child-OIDP has been validated by Yusof and Jaafar in 2012 to assess OHRQoL among 11–12 year old Malay-speaking children in Malaysia [17]. The scoring system of the index directly relates perceived oral health needs with the severity of impact scores. The index asks questions about oral problems in the past 3 months, and whether the problems had impacted on 8 daily performances, i.e. eating, speaking, cleaning teeth, relaxing (including sleeping), smiling (including laughing and showing teeth without embarrassment), maintaining emotional stability, studying (including going to school and doing homework), and socialising. It was initially tested for face validity on 20, 11–12 year old OA children before the conduct of the study. The face validity assessment required the OA children to answer the index individually followed by a group discussion to assess their understanding on the purpose of the index, the instructions, the wording of items, and the response options. At the end of the discussion, it was concluded that the children understood the items well. No changes were made to any of the items.

For oral health examination, dental caries was scored using the decayed, missing, and filled tooth index for both primary and permanent teeth (dft/DMFT) according to the World Health Organization’s criteria [19]. The periodontal status was assessed using the Community Periodontal Index (CPI) on six index teeth [20] and the gingival health status was assessed using the Gingival Bleeding Index (GBI) [21].

Prior to data collection, 3 examiners were calibrated in oral examination using the dft/DMFT indices, the CPI, and the GBI. During the calibration exercise, 10 schoolchildren aged 11–12 years were examined by a dental specialist using the indices above whose scores were set as the gold standard. Cohen’s kappa values for inter-examiner and intra-examiner reliability suggested good agreement. Cohen’s kappa values for inter-examiner reliability were 0.87 for dft/DMFT, 0.76 for CPI, and 0.71 for GBI. Cohen’s kappa values for intra-examiner reliability were 0.8 for dft/DMFT, 0.79 for CPI, and 0.76 for GBI, respectively.

### Conduct of study

Ethical approval for the study was granted by the Medical Ethics Committee, Faculty of Dentistry, University of Malaya [Ref No. DF DP1410/0074(P)]. Permission to conduct the study was obtained from the Ministry of Education, the Orang Asli Welfare Department, Pahang State Education Department, CH District Education Department, and the respective schools.

Prior to data collection, consent for the OA children to participate in the study was obtained from OA parents or guardians including questions on parent’s education, occupation, and household income. On data collection day, all OA children with consent were assembled in their respective classroom. The researchers distributed the questionnaire to the OA children and supervised the answering process with assistance from the class teacher.

After completing the questionnaire, the OA children underwent an oral examination carried out by the calibrated examiners. The examination was carried out with the children seated on a portable dental chair under natural daylight. Dental caries was scored using the dft/DMFT indices [20] while periodontal status was assessed using the CPI on six index teeth [21, 22].

### Data analysis

Data were entered and analysed using the Statistical Package for Social Sciences (SPSS) software version 23.0. Oral impacts on daily performances (OIDP) scores were described in terms of prevalence, impact score, impact intensity, and extent of impact. The overall prevalence of oral impacts was calculated as the percentage of children with OIDP score greater than zero, i.e. at least one daily performance affected by oral condition(s). Item prevalence was calculated as the percentage of children with OIDP score greater than zero on that performance [23].

Impact score of a performance was calculated by multiplying the severity and frequency of impact to give a score of 0 to 9 (0 = no impact, 9 = maximum impact). Total OIDP score was calculated by summing the scores of the eight daily performances while total percentage score was calculated by dividing the total score by 72, and multiplied by 100 [23]. Therefore, total percentage score ranges from 0 to 100 where a higher score indicates higher oral impact and poorer OHRQoL, and vice versa.

Impact intensity refers to the percentage of children with different levels of impact on each of the 8 performances. The categories are ‘no impact’ (score = 0), ‘very little’ (score = 1), ‘little’ (score = 2), ‘moderate’ (score = 3–4), ‘severe’ (score = 6), and ‘very severe’ (score = 9) levels of impact intensity. Finally, extent of impact was measured by calculating the number of performances with impacts (PWI) affecting a child’s quality of life. The value ranged from 0 to 8 PWI.

The OIDP scores were described by socio-demographic factors, oral health behaviours and clinical parameters. In this study, the distribution of OIDP scores was skewed. Therefore, non-parametric statistics, i.e. the Kruskal-Wallis and Mann Whitney tests were used to compare OIDP scores within groups with level of significance set at *p* < 0.05. Post hoc test was carried out to identify significant differences within group with 3 or more categories; the Dunn Bonferoni and Mann Whitney tests were used with level of significance set at *p* < 0.017. Multiple logistic regression was carried out to identify factors associated with oral impacts among the OA children.

## Results

In total, 227 of 249 OA children responded with 91.2% response rate. Twenty students were absent from school on data collection day and were excluded from the study. Only two (2) parents (0.8%) did not give consent for their children to participate in the study. The proportion of male children was slightly higher (51.5%) than female children (48.5%). All children were from the *Semai* tribe. The majority aged 11 years (66.5%) while 33.5% aged 12 years. Most fathers (61.7%) and mothers (62.6%) had education up to primary school level or higher. The majority of fathers worked in agriculture (55.5%) while the majority of mothers were housewives (59.5%). Less than half (45.4%) had family income <RM1000 per month (Table 1).

**Table 1:** Frequency distribution of OA children’s sociodemographic characteristics, oral health-related behaviours, oral health status, and their associations with Child-OIDP score

Variable	n (%)	OIDP score Mean (SD)	OIDP score (Quartiles)	*P* value^1^
Age/year				
11	151 (66.5)	6.38 (9.7)	(0.0, 2.8, 8.3)	0.318^2^
12	76 (33.5)	3.73 (5.1)	(0.0, 2.8, 5.6)	
Gender				
Female	110 (48.5)	5.63 (8.1)	(0.0, 2.8, 7.3)	0.535^2^
Male	117 (51.5)	5.37 (8.9)	(0.0, 1.4, 6.3)	
Father’s education*				
No education	65 (28.6)	6.52 (7.8)	(0.0, 4.2, 11.1)	
Primary school	40 (17.6)	5.31 (9.4)	(0.0, 1.4, 8.0)	0.125
Secondary school or higher	100 (44.1)	4.71 (8.4)	(0.0, 2.1, 5.6)	
Mother’s education*				
No education	65 (28.6)	4.85 (6.3)	(0.0, 2.8, 6.9)	
Primary school	56 (24.7)	5.56 (7.0)	(0.0, 2.8, 8.3)	0.436
Secondary school or higher	86 (37.9)	5.79 (10.7)	(0.0, 1.4, 5.6)	
Father’s occupation*				
Salaried occupation	76 (33.5)	4.22 (7.7)	(0.0, 2.1, 5.6)	
Self-employed/Agriculture-based	126 (55.5)	6.58 (9.4)	(0.0, 2.8, 8.7)	0.229
No occupation	18 (7.9)	4.39 (5.7)	(0.0, 2.1, 6.9)	
Mother’s occupation*				
Salaried occupation	10 (4.4)	4.31 (4.0)	(2.1, 2.8, 6.6)	
Self-employed/Agriculture-based	80 (35.2)	6.21 (8.7)	(0.0, 2.8, 9.4)	0.509
No occupation	135 (59.5)	5.22 (8.7)	(0.0, 1.4, 5.6)	
Household income*				
≤ RM1000	103 (45.4)	6.30 (9.7)	(0.0, 2.7, 8.3)	0.238^2^
> RM1000	31 (13.7)	7.21 (9.1)	(0.0, 4.2, 9.7)	
Frequency of toothbrushing				
≥ 2x/day	190 (83.7)	5.10 (7.9)	(0.0, 2.8, 6.9)	0.325^2^
< 2x/day	37 (16.3)	7.51 (11.1)	(0.0, 2.8, 9.7)	
Frequency of using toothpaste				
≥ 2x/day	182 (80.2)	5.39 (8.3)	(0.0, 2.8, 8.3)	
< 2x/day	43 (18.9)	6.07 (9.5)	(0.0, 2.8, 5.6)	0.831
Never	2 (0.9)	2.08 (2.9)	(0.0, 2.1, −)	
Frequency of cigarette smoking				
≥ Once a week	9 (4.0)	2.47 (3.3)	(0.0, 1.4, 4.2)	0.433^2^
Never	218 (96.0)	5.62 (8.7)	(0.0, 2.8, 6.9)	
Frequency of betel nut chewing				
≥ 1x/day	153 (67.4)	5.19 (8.5)	(0.0, 1.4, 6.9)	0.036
1–3 times/week	32 (14.1)	8.85 (10.5)^β^	(1.4, 4.2, 13.9)	
Once a month or never	42 (18.5)	4.03 (6.4)^β^	(0.0, 1.4, 5.6)	
Frequency of mouth rinsing with water after eating				
> 3x/day	52 (22.9)	5.64 (7.4)	(0.0, 2.8, 8.3)	
1–3 times a day	120 (52.9)	4.57 (6.6)	(0.0, 2.8, 5.6)	0.932
3 times a week or less	18 (7.9)	5.48 (8.8)	(0.0, 1.4, 7.3)	
Never	37 (16.3)	8.30 (13.7)	(0.0, 1.4, 11.1)	
Frequency of dental examination				
Once a year	222 (97.8)	5.57 (8.6)	(0.0, 2.8, 6.9)	0.354^2^
Never	5 (2.2)	1.94 (2.7)	(0.0, 0.0, 4.9)	
DMFT score				
0	87 (38.3)	3.88 (7.6)^α^	(0.0, 0.0, 4.2)	
1–3	117 (51.5)	6.11 (8.4)^α^	(0.0, 2.8, 8.3)	0.024
> 3	23 (10.1)	8.76 (11.4)	(0.0, 2.8, 15.3)	
DT score				
0	95 (41.9)	4.09 (7.6)	(0.0, 1.4, 5.6)	
1–3	114 (50.2)	5.81 (8.3)	(0.0, 2.8, 8.3)	0.093
> 3	18 (7.9)	10.88 (12.1)	(0.0, 6.9, 19.4)	
dft score				
0	133 (58.6)	4.27 (4.2)^α^	(0.0, 1.4, 5.6)	
1–3	76 (33.5)	6.76 (9.7)^α^	(0.0, 4.2, 8.3)	0.005
> 3	18 (7.9)	9.18 (10.6)	(0.0, 6.3, 14.6)	
GBI				
No	9 (4.0)	3.85 (3.09)	(1.4, 2.8,5.6)	0.558^2^
Yes	218 (96.0)	5.56 (8.67)	(0.0, 2.8, 6.9)	
CPI Score				
CPI 0 (Healthy)	9 (4.0)	3.85 (3.1)	(0.7, 2.8, 5.6)	
CPI 1 (Bleeding on probing)	76 (33.4)	4.62 (7.8)	(0.0, 2.8, 12.9)	0.334
CPI 2 (Calculus)	142 (62.6)	6.06 (9.2)	(0.0, 3.5, 11.1)	

^1^ Kruskal Wallis test, significant level is *p* < 0.05

^2^ Mann Whitney test, significant level is *p* < 0.05

^α^ Mann Whitney test, adjusted significant level *p* < 0.017

^β^ Dunn Bonfferoni Post Hoc test, significant level *p* < 0.017

* number does not equal to 227 due to missing data

In terms of self-reported oral health-related behaviours, majority of the OA children brushed their teeth ≥ 2x/day (83.7%) and used toothpaste ≥ 2x/day (80.2%). Only a few children (4.0%) smoked cigarettes ≥ 1/week. More than two-thirds chewed betel nut ≥ 1/day (67.4%) while a small minority chewed 1–3 times/week (14.1%). Over half of the OA children (52.9%) rinsed their mouth 1–3 times/day after meal while over one-fifth (22.9%) rinsed >3x/day. The majority had a dental examination by the visiting dental therapist once a year (97.8%). Only 2.2% never had a dental examination.

In terms of oral health status, over three-fifths of the OA children (61.6%) had dental caries in permanent teeth (mean DMFT = 1.36), 58.1% had active decay in permanent teeth (DT) while over two-fifths (41.4%) had dental caries in primary teeth (mean dft = 1.01). A large majority (96.0%) of the OA children had gingivitis (GBI = yes). Further to that, 62.6% had CPI Score 2 and 33.4% had CPI Score 1. Only a few children (4.0%) had healthy gingiva (CPI Score 0).

No difference in OIDP scores by socio-demographic background was observed. In terms of the association between OIDP scores and oral health behaviours, an association was observed between the frequency of betel nut chewing and OIDP scores (*p* = 0.036), i.e. mean OIDP score was lower in children who chewed betel nut once a month or less compared with children who chewed 1–3 times/week (*p* < 0.017).

In terms of the association between OIDP scores and oral health status, an associations between dental caries experience (DMFT, dft) and OIDP scores were observed, respectively (*p* < 0.050), i.e. as the DMFT/dft scores increased, the mean OIDP scores were also increased. Within-group comparison showed that children with DMFT score of 1–3 had a higher mean OIDP score compared with caries-free children (*p* < 0.050). Similar association was also observed between mean OIDP score of those with dft score of 1–3 and mean OIDP score of caries-free children.

The overall prevalence of oral impacts was 58.6%, i.e. about 6 in 10 children had at least 1 oral impact in the past 3 months (Table 2). In terms of daily performances, the highest prevalence of oral impact was reported on eating (35.2%), followed by cleaning teeth (22.0%), relaxing (15.9%), and emotional stability (15.9%). Oral impacts on speaking (10.1%) and doing homework (11.0%) were reported to be among the lowest. The mean OIDP total score was 5.45 (SD = 8.5). Mean OIDP score for each performance, from the highest to the lowest, were related to eating (0.35, SD = 0.5), cleaning teeth (0.22, SD = 0.4), relaxing and emotional stability (0.16, SD = 0.4), smiling (0.15, SD = 0.4), socialising (0.14, SD = 0.4), doing homework (0.11, SD = 0.3), and speaking (0.10, SD = 0.3).

**Table 2:** Prevalence, score and intensity of oral impacts on daily performances among OA schoolchildren in Cameron Highlands (*n* = 227)

Performance									
Oral impacts on daily performances	Overall impact n (%)	Eating n (%)	Speaking n (%)	Cleaning teeth n (%)	Relaxing n (%)	Emotion n (%)	Smiling n (%)	Doing Homework n (%)	Socialising n (%)
Prevalence (%)	133 (58.6)	80 (35.2)	23 (10.1)	50 (22.0)	36 (15.9)	36 (15.9)	34 (15.0)	25 (11.0)	32 (14.1)
Impact score									
Range^a^	0–100	0–9	0–9	0–9	0–9	0–9	0–9	0–9	0–9
Mean (SD)	5.45 (8.5)	0.35 (0.5)	0.10 (0.3)	0.22 (0.4)	0.16 (0.4)	0.16 (0.4)	0.15 (0.4)	0.11 (0.3)	0.14 (0.4)
Percentiles	(0.0,2.8,6.9)	(0.0,0.0,1.0)	(0.0,0.0,0.0)	(0.0,0.0,0.0)	(0.0,0.0,0.0)	(0.0,0.0,0.0)	(0.0,0.0,0.0)	(0.0,0.0,0.0)	(0.0,0.0,0.0)
Impact intensity [n (%) of children with impacts]									
Very little	53(23.3)	20 (9.2)	8 (3.7)	7 (3.2)	5 (2.3)	15 (6.9)	5 (2.3)	7 (3.2)	13 (6.0)
Little	71 (31.3)	31 (14.2)	8 (3.7)	19 (8.7)	16 (7.3)	13 (6.0)	10 (4.6)	9 (4.1)	8 (3.7)
Moderate	54 (23.8)	24 (11.0)	5 (2.3)	15 (6.9)	10 (4.6)	5 (2.3)	9 (4.1)	4 (1.8)	8 (3.7)
Severe	18 (7.9)	5 (2.3)	1 (0.5)	3 (1.4)	3 (1.4)	3 (1.4)	9 (4.1)	1 (0.5)	2 (0.9)
Very Severe	14 (6.2)	0 (0)	1 (0.5)	6 (2.6)	2 (0.9)	0(0)	1 (0.5)	4 (1.8)	1 (0.5)

^a^ Maximum score for each performance = 9; Maximum score for the eight performances = 100

In terms of impact intensity, the majority had ‘very little’ to ‘moderate’ levels of impact intensity on the 8 daily performances (78.4%). Only 14.1% reported having oral impacts of ‘severe’ to ‘very severe’ levels of impact intensity. For each performance, more children reported having ‘very little’ to ‘moderate’ levels of impact intensity. Smaller proportions of OA children reported having ‘severe’ to ‘very severe’ levels of impact intensity on smiling (4.6%), cleaning teeth (4.0%), eating (2.3%), relaxing (2.3%) and doing homework (2.3%). In terms of PWI, 90.2% of the OA children had up to 4 PWI, i.e. 39.8% had 1 PWI, 30.1% had 2 PWI, 10.5% had 3 PWI, and 9.8% had 4 PWI.

The most frequently reported oral problem causing oral impact was ‘bleeding gum’ (66.2%), followed by ‘toothache’ (56.4%), and ‘fractured tooth’ (52.6%) while the least frequently reported oral problem causing oral impact was ‘cleft lip/palate’ (4.5%) (Table 3).

**Table 3:** Frequency of oral conditions perceived to have caused overall oral impact (*n* = 133)

Oral Conditions	Oral conditions perceived to have caused overall impact, n (%)
Toothache	75 (56.4)
Sensitive tooth	67 (50.4)
Tooth decay/ Hole in the tooth	44 (33.1)
Loose primary tooth	44 (33.1)
Spacing between teeth	23 (17.3)
Fractured tooth	70 (52.6)
Colour of teeth	64 (48.1)
Shape or size of teeth	32 (24.1)
Position of teeth	31 (23.3)
Bleeding gum	88 (66.2)
Swollen gum	29 (21.8)
Plaque and/or calculus	69 (51.9)
Ulcer	40 (30.1)
Bad breath	58 (43.6)
Cleft lip/ cleft palate	6 (4.5)
Erupting permanent tooth	69 (51.9)
Missing permanent tooth	10 (7.5)

* Subjects can answer more than one oral condition perceived to have caused overall oral impact

Table 4 shows findings from multiple logistic regression analysis to determine factor(s) associated with oral impact in the past 3 months. Of the 4 factors included in the analysis (DMFT score, DT score, dft score, and frequency of betel nut chewing), only one factor, i.e. dft score was found to be associated with oral impact when other factors were controlled (*p* < 0.050). Children with dft score of 1–3 are 2.56 times more likely to experience at least 1 oral impact on the 8 daily performances compared with children with no caries in the primary teeth. Children with dft > 3 are 7.53 times more likely to experience oral impact in the past 3 months compared with children with no caries in the primary teeth (*p* = 0.064)*.*

**Table 4:** Associated factor for the presence of oral impact (Child-OIDP> 0) among the OA children (results of Multiple Logistic Regression analysis)

Variable	Regression coefficient B(SE)	Wald statistic	95% CI for Adjusted Odds Ratio^a^			*P* value
			Lower	OR	Upper	
dft score						
0	–	–		1.00		
1–3	0.94 (0.40)	5.56	1.17	2.56	5.57	0.018^b^
> 3	2.02 (1.09)	3.44	0.89	7.53	63.62	0.064

Hosmer-Lomeshow test (*p* = 1.000), classification table (overall correctly classified percentage = 63.0%)

^a^ Forward LR Multiple Logistic Regression model was applied

^b^ *p* value < 0.05

## Discussion

This is the first study carried out in Malaysia to assess the oral health status, oral health-related behaviours, and OHRQoL among 11–12 year old OA children in Cameron Highlands, Malaysia.

Overall, the study showed that the prevalence of caries and periodontal disease among the OA children in the sample was high with high prevalence of oral impacts. Caries in primary teeth was found to be associated with children’s OHRQoL in the multivariate analysis. The study also showed that the majority of OA children chewed betel nuts on daily basis.

The findings from this study showed that the overall prevalence of oral impact was 58.6% with mean OIDP score was 5.45 (SD = 8.5). This prevalence was lower than the prevalence in a similar study conducted in urban schools in Kuala Lumpur where 67.7% of 11–12 year old children had oral impacts on their daily activities [17].The relatively lower prevalence of oral impacts in the OA children sample could be due to their diet where it consists mostly of staple foods with low in sugars but high in fibres [24]. They also have less exposure to sugary food and drinks in rural communities and in schools compared to children in urban areas. Therefore, they are less likely to suffer from caries-related oral impacts due to toothache and discomfort.

The prevalence of oral impact in the current study was lower than that reported in similar studies in other countries namely Thailand (85.2%) [23], France (73.2%) [13], India (60%) [25], Brazil (80.7%) [11], and Italy (94.5%) [24]. However, the prevalence of oral impact in the current study was higher than that reported in the UK (40.4%) [27], Tanzania (28.6%) [12], Spain (36.5%) [26], Sudan (54.6%) [14], and South Africa (36.2%) [29]. Differences in oral health awareness, socio-cultural values, dietary patterns, and oral hygiene behaviours may explain the differences in oral impact prevalence between children in different countries.

In this study, half of those with impacts suffered ‘very little’ to ‘little’ levels of impact intensity. Among the 8 daily performances, the ability to eat properly was reported as the most affected performance. Similar finding was also reported in other studies using the Child OIDP index [12–14, 17, 26, 27]. Only one study reported differently where the ability to clean teeth was the most affected performance [14]. In rural OA communities, the ability to eat properly is of great importance in order to provide energy for daily activities as they spend most of their time outdoors. Bleeding gum, toothache, fractured tooth, exfoliating primary teeth, erupting permanent teeth, and cavities were the common oral conditions cited in this study that prevented the children from eating their food properly which subsequently affected their quality of life. Bleeding gum may interfere with the taste of food and drinks while toothache may get worse when biting down food or taking hot or cold drinks.

The second most prevalent oral impact reported in this study was the ability to clean teeth where 2.6% suffered ‘very severe’ impact. The inability to clean the teeth properly could be due to bleeding gums which was the most frequently reported factor to have caused oral impacts among the OA children. Gingival bleeding is a common consequence of gingival inflammation, and children with this problem may face difficulties in brushing their teeth effectively [28]. Lack of emphasis on the importance of good oral hygiene, poor oral health knowledge, poor oral hygiene practice and non-compliance to oral self-care to prevent gingivitis and its progression to periodontitis are some of the possible reasons. Therefore, a long term educational intervention is required to improve their oral health knowledge, attitudes and oral hygiene behaviours. Most children are unaware if they have periodontal problem especially at early stage as it rarely presents with any clinical symptom until the gingiva started to bleed during toothbrushing [30].

Toothache was reported as the second most common oral condition affecting the OA children’s daily performances. This is not surprising because children suffering from toothache would become substantially disadvantaged compared to their healthy peers [31]. Apart from toothache, caries may also affect children’s quality of life by causing discomfort, facial disfigurement, eating difficulties, and sleep disturbances as well as a higher risk of hospitalisation, high treatment costs, and loss of school days [32]. The finding of the current study was also supported by Jackson et al. [33] where children with poor oral health were almost three times more likely to miss school days as a result of dental pain and were indirectly implicated on their school performances.

In this study, betel nut chewing habit was significantly associated with children’s OHRQoL. Children who chewed betel nut regularly had severe oral impact compared to those who chewed betel nut irregularly. One of the possible impacts from chewing betel nut is poor discolouration of teeth and gingiva due to the extrinsic staining particularly in those with poor tooth brushing practice [34]. Tooth discolouration could impact the children in terms of their ability to smile and socialise without embarrassment. Despite the habit is a known risk factor for oral cancer [34], it is highly prevalent among OA populations in Malaysia [35]. As a preventive measure, the school dental team should involve further than giving oral health education in school. A healthy policy to ban betel nut chewing in school should be introduced. This should be supported by the teachers, school, and the community. Extending the preventive effort beyond the school compound to include the community is also recommended. This is because betel nut chewing habit is passed down to children from their parents, relatives, and the community members. Thus, efforts to tackle the problem at community level must take into account the socio-cultural context of betel nut chewing habit in the OA community. To be effective, the health promotion principles should be applied to empower the community, change social norms, and address the socio-cultural barriers for change. An active participation from village elders and tribal leaders is essential for the success of the programme [36].

Caries in primary teeth has emerged as the only factor in the multiple logistic regression model associated with oral impact in the past 3 months. A possible explanation for this is that OA children with caries in primary teeth would be more likely to suffer from dental pain and discomfort that will affect their ability to chew food, speak well, clean their teeth, sleep, relax, smile, perform school work, and socialise with friends [33, 37]. In addition, caries in primary teeth had been shown to have impacts on parents by means of expensive dental treatment, travelling expenses, and loss of working days [31, 38].

Based on the findings of this study, concerted efforts to improve the OA children’s OHRQoL must address the caries problem in the primary teeth which means future preventive efforts must target a younger age group children with the aim to improve oral hygiene practices and reduce sugary food and drinks consumption in order to reduce caries in primary teeth. If this is feasible, the long term effect may also be seen in lowering the caries prevalence in the permanent dentition as the children grow older. This approach should take precedence among dental policy-makers. In the current situation, the incremental dental care service provided by the Oral Health Programme, Ministry of Health, includes an oral examination, a treatment provision, and an oral health education for OA school children 6 years and older. It is recommended that a dental outreach program be expanded to include a community-based oral health promotion programme targeting younger OA children below 6 years and their parents. The aim is to empower OA parents with knowledge and skills so they can improve their children’s oral hygiene practice and to provide improved facilities for oral health in the community setting. In areas where preschool centres for 4–6 year old children are available, a collaboration with the school teachers to include regular oral health education in the class lessons is recommended. In-school tooth brushing exercise using fluoridated toothpaste is also recommended with supervision from the class teacher.

This study had a limitation in that the total number of participations did not reach the calculated sample size of *n* = 342. However, as most OA communities reside in remote areas, the findings of this study could be said to reflect the OA children of similar age group in Pahang state due to their similar socio-cultural characteristics. Future research should consider assessing the impact of a programme to promote positive oral hygiene practices among younger OA children and the feasibility of active participation of teachers and parents in oral health promotion programmes in schools as well as in community settings.

## Conclusions

The 11–12 year old OA children in Cameron Highland had high prevalence of caries and gingivitis with the majority chewed betel nut regularly. Caries in primary teeth is associated with poor OHRQoL. Future programmes should target younger age group children to promote positive oral hygiene practices, reduce caries, and improve quality of life.

## Data Availability

As more articles are to be published, research data will not be made available.

## Abbreviations

CH: Cameron Highlands
Child-OIDP: Child Oral Impacts on Daily Performances
CPI: Community Periodontal Index
dft: Decayed filled teeth
DMFT: Decayed Missing Filled Teeth
DT: Decayed teeth
GBI: Gingival Bleeding Index
OA: *Orang Asli*
OHRQoL: Oral health-related quality of life
OIDP: Oral Impacts on Daily Performances
PWI: Performances With Impacts
SD: Standard Deviation
SPSS: Statistical Package for Social Sciences
